# Role of C-reactive Protein and Procalcitonin in Early Diagnostic Accuracy and Their Prognostic Significance in Sepsis

**DOI:** 10.7759/cureus.70358

**Published:** 2024-09-27

**Authors:** Muhammad Daud, Mir Baz Khan, Qudrat Ullah Qudrat, Izhar Ullah, Sheheryar Khan, Muhammad Zubair Khan, Ihtesham Yousuf, Faizan Ahmad

**Affiliations:** 1 General Surgery, Lady Reading Hospital, Peshawar, PAK; 2 Emergency Medicine, Bacha Khan Medical Complex, Peshawar, PAK; 3 Medicine, Naseer Teaching Hospital, Peshawar, PAK; 4 Medicine, Hayatabad Medical Complex Peshawar, Peshawar, PAK; 5 Internal Medicine, District Head Quarter Teaching Hospital/GMC, Dera Ismail Khan, PAK; 6 Internal Medicine, Lady Reading Hospital, Peshawar, PAK; 7 Internal Medicine, Khyber Teaching Hospital, Peshawar, PAK; 8 Internal Medicine, Hayatabad Medical Complex Peshawar, Peshawar, PAK

**Keywords:** c-reactive protein, diagnostic accuracy, intensive care unit, procalcitonin, prognostic significance, sepsis

## Abstract

Introduction: Sepsis is a serious condition that often results in high fatality rates, particularly in intensive care units (ICUs). Its nonspecific clinical characteristics makes early diagnosis and therapy difficult, despite how critical they are. The use of biomarkers like procalcitonin (PCT) and C-reactive protein (CRP) in the diagnosis and prognosis of sepsis has demonstrated encouraging results. In contrast to PCT, which is highly selective for bacterial infections, CRP is an acute-phase protein that reflects systemic inflammation.

Objective: This study aimed to assess the diagnostic accuracy and prognostic significance of CRP and PCT in early sepsis detection and outcome prediction.

Methodology: This study was a retrospective cohort study that involved 90 patients in the ICU who met the criteria for sepsis-3. CRP and PCT levels, clinical data, and outcomes were obtained from electronic medical records. The diagnostic accuracy was tested using receiver operating characteristic (ROC) curves, while the prognostic relevance was analyzed by Kaplan-Meier survival analysis and Cox proportional hazards regression.

Results: The mean CRP level was 102.3 mg/L and PCT level was 5.4 ng/mL. ROC analysis revealed an area under the curve (AUC) of 0.78 for CRP and 0.82 for PCT, indicating better diagnostic performance for PCT. High levels of CRP and PCT were associated with poorer survival, with median survival times of 18 and 15 days, respectively, for high-level groups. Cox regression identified CRP and PCT as significant predictors of mortality, with hazard ratios of 1.50 and 1.68, respectively.

Conclusion: Both CRP and PCT are valuable biomarkers for diagnosing and prognosticating sepsis. PCT, with its higher specificity for bacterial infections, demonstrates superior diagnostic accuracy compared to CRP. Elevated levels of both biomarkers are associated with increased mortality risk, highlighting their potential role in early sepsis management and outcome prediction.

## Introduction

Sepsis, a potentially lethal syndrome caused by an uncontrolled immune response to infection, continues to be a significant cause of illness and death globally. Despite the advancements made in medical care, sepsis still has significant death rates, especially in intensive care units (ICUs), where patients frequently have severe and fast advancing forms of the syndrome [1]. Timely identification and prompt response are crucial for enhancing outcomes in sepsis; nevertheless, the lack of specificity in its clinical manifestation presents substantial difficulties [2]. As a result, there is a continuous effort to find dependable biomarkers that might assist in the timely detection and prediction of sepsis. Out of the several biomarkers that have been examined, C-reactive protein (CRP) and procalcitonin (PCT) have shown great potential as candidates for sepsis due to their connection with the inflammatory and infectious processes that cause it [3].

CRP is an acute-phase protein synthesized by the liver in response to inflammation. Its levels rise significantly during systemic infections, making it a widely used marker of inflammation in clinical practice. The role of CRP in sepsis has been extensively studied, and it is well-established that elevated CRP levels correlate with the presence of infection and the severity of the inflammatory response. However, CRP is a non-specific marker, as its levels can also be elevated in non-infectious inflammatory conditions such as trauma, surgery, and chronic inflammatory diseases. This lack of specificity limits the utility of CRP as a standalone diagnostic tool in sepsis, highlighting the need for additional markers to enhance diagnostic accuracy [4,5].

Procalcitonin, a precursor of the hormone calcitonin, is another biomarker that has gained considerable attention in the context of sepsis. Unlike CRP, PCT is more specifically associated with bacterial infections, and its levels increase significantly in response to systemic bacterial infections, including sepsis. The specificity of PCT for bacterial infections is thought to be due to its induction by bacterial endotoxins and pro-inflammatory cytokines, while its production is suppressed in viral infections and non-infectious inflammatory conditions. This specificity has positioned PCT as a potentially superior biomarker for differentiating bacterial sepsis from other causes of systemic inflammation. Furthermore, PCT levels have been shown to correlate with the severity of sepsis and patient outcomes, making it a valuable tool not only for diagnosis but also for prognostication [6,7].

The utility of CRP and PCT as biomarkers in sepsis has been the subject of numerous studies, which have generally supported their roles in early diagnosis and risk stratification [3,8,9,10]. However, the relative strengths and limitations of each marker, as well as their combined use, continue to be areas of active investigation. Some studies have suggested that PCT may offer greater diagnostic accuracy than CRP due to its higher specificity for bacterial infections [10], while others have highlighted the value of CRP as a marker of inflammation that can complement PCT in the diagnostic process [11,12]. The prognostic significance of these markers, particularly in relation to established clinical scoring systems like the Sequential Organ Failure Assessment (SOFA) score, remains an important area of research [13].

In recent years, efforts to improve the management of sepsis have focused on the development of early warning systems and diagnostic algorithms that incorporate biomarkers like CRP and PCT. These approaches aim to enable earlier detection of sepsis, prompt initiation of appropriate therapies, and improved risk stratification, ultimately leading to better patient outcomes. However, the optimal use of CRP and PCT in clinical practice, including the thresholds for intervention and the interpretation of serial measurements, is still being refined. Moreover, the potential for these biomarkers to guide therapeutic decisions, such as the initiation and discontinuation of antibiotics, is an area of considerable interest.

Objective

The objective of this study was to evaluate the early diagnostic accuracy and prognostic significance of CRP and PCT in patients with sepsis.

## Materials and methods

Study design and population

The current research was conducted as a retrospective cohort study aimed at assessing the diagnostic accuracy and prognostic importance of CRP and PCT in sepsis within a hospital setting. The research cohort included adult individuals aged 18 years and above who were hospitalized to the ICU with a suspected or confirmed diagnosis of sepsis, specifically due to underlying conditions such as pneumonia, urinary tract infections, abdominal infections, and skin infections. The patients included in the study were required to meet the sepsis-3 criteria for sepsis or septic shock within 24 hours of being admitted. Exclusion criteria were applied to patients with chronic inflammatory diseases (e.g., rheumatoid arthritis, lupus), those who had undergone surgery within the past 30 days, patients on ongoing immunosuppressive therapy, and those with end-stage renal or liver disease, as these conditions could influence CRP and PCT levels.

Sample size calculation

Ninety volunteers were the target number for the research. Instead of using a formal sample size calculation, the sample size was determined by considering feasibility in light of the limitations of the accessible population and the retrospective character of the investigation. It was decided that this sample size would be adequate to offer some initial insights into the prognostic and diagnostic value of PCT and CRP in sepsis.

Data collection

Retrospective data collection was done using electronic medical records. This comprised patient outcomes (survival, length of ICU stay), laboratory findings (CRP, PCT, white blood cell count, lactate), clinical parameters (heart rate, blood pressure, temperature), and demographic information (age, gender). At the time of admission (baseline), as well as every 24 hours for the first 72 hours, CRP and PCT levels were measured. The main outcome variables were the predictive value of PCT and CRP in predicting 28-day mortality and their diagnostic accuracy in identifying sepsis. The length of an ICU stay, organ failure rates, and SOFA scores were examples of secondary factors.

Laboratory analysis

CRP levels were measured using a high-sensitivity immunoturbidimetric assay, which had a detection limit of 0.1 mg/L and a coefficient of variation (CV) of less than 5%. PCT levels were determined using an electrochemiluminescence immunoassay, with a detection threshold of 0.02 ng/mL and a CV of less than 3%. These assays were performed in the hospital's central laboratory under stringent quality control protocols. Calibration curves were established for each assay, and internal controls were regularly employed to ensure assay accuracy and precision.

Statistical analysis

IBM SPSS Statistics for Windows, version 27.0 (IBM Corp., Armonk, NY) was used for statistical analysis. Descriptive statistics were employed to succinctly summarize the fundamental features of the subjects. The diagnostic precision of CRP and PCT in detecting early sepsis was evaluated by the receiver operating characteristic (ROC) curve analysis, and the area under the curve (AUC) was computed for each biomarker. The study assessed the sensitivity, specificity, positive predictive value (PPV), and negative predictive value (NPV) for different threshold values of CRP and PCT. Survival analysis using the Kaplan-Meier method was conducted to assess the prognostic importance of CRP and PCT. The survival results were then compared depending on the levels of these biomarkers. The log-rank test was employed to evaluate disparities in survival curves, while a multivariate Cox proportional hazards regression analysis was performed to uncover autonomous predictors of death, while accounting for confounding variables such as age, gender, SOFA score, and comorbidities.

Ethical considerations

The study protocol received approval from the Institutional Review Board (IRB) of Lady Reading Hospital-MTI, Peshawar. Given the retrospective nature of the study, informed consent was waived, but patient confidentiality was strictly maintained. All patient data were anonymized before analysis to protect privacy and confidentiality.

## Results

The study population had a mean age of 65.4 years (SD: 12.7), with ages ranging from 34 to 89 years. Males constituted 54 participants, representing 60% of the total population, while females made up 36 participants, accounting for 40%. The mean SOFA score was 7.0, with a median of 7.0 (SD: 3.1), indicating moderate organ dysfunction. For participants with sepsis, the mean CRP level was 98.5 mg/L (SD: 38.5), while those with septic shock had a mean CRP of 115 mg/L (SD: 38.5), with a significant p-value of 0.001. The mean PCT levels were 4.8 ng/mL for sepsis and 7.0 ng/mL for septic shock (SD: 2.3), both showing significant differences (p < 0.001). The mean white blood cell count was 15.8 ×10⁹/L (SD: 6.3), and the mean lactate level was 3.2 mmol/L (SD: 1.8). The average length of ICU stay was 10.4 days (SD: 7.2), with a median of 8 days. The 28-day mortality rate was 35%, corresponding to 31 participants, with septic shock being the cause of death in 48.4% of cases. Other causes included multi-organ failure (32.3%), acute respiratory distress syndrome (9.7%), and cardiac arrest (9.7%), as shown in Table 1.

**Table 1:** Study population and baseline characteristics SOFA Score: Sequential Organ Failure Assessment score; CRP (mg/L): CRP (mg/L); PCT (ng/mL): PCT (nanograms per milliliter); white blood cell count (×10⁹/L): white blood cell count (billions per liter); lactate (mmol/L): lactate (millimoles per liter)

Variable		Mean (sepsis)	Mean (septic shock)	Standard deviation	Minimum	Maximum	n (%)	p-value
Age (years)		64	68.5	12.7	34	89	-	0.12
Gender (Male)		36 (60%)	18 (60%)	-	-	-	54 (60%)	-
Gender (Female)		24 (40%)	12 (40%)	-	-	-	36 (40%)	
SOFA Score		6.5	9	3.1	2	15	-	<0.001
CRP (mg/L)		98.5	115	38.5	12	240	-	0.001
PCT (ng/mL)		4.8	7	2.3	0.5	12.8	-	<0.001
WBC (×10⁹/L)		12.5	15.0	6.3	4.1	32.7	-	0.15
Lactate (mmol/L)		2.8	4.5	1.8	0.5	8.7	-	0.25
Length of ICU stay (days)		9.0	12.3	7.2	2	30	-	0.18
28-day mortality		-	-	-	-	-	31 (35%)	-
Cause of mortality	Septic shock	-	-	-	-	-	15 (48.4%)	-
	Multi-organ failure	-	-	-	-	-	10 (32.3%)	-
	Acute respiratory distress syndrome	-	-	-	-	-	3 (9.7%)	-
	Cardiac arrest	-	-	-	-	-	3 (9.7%)	-

**Figure 1:**
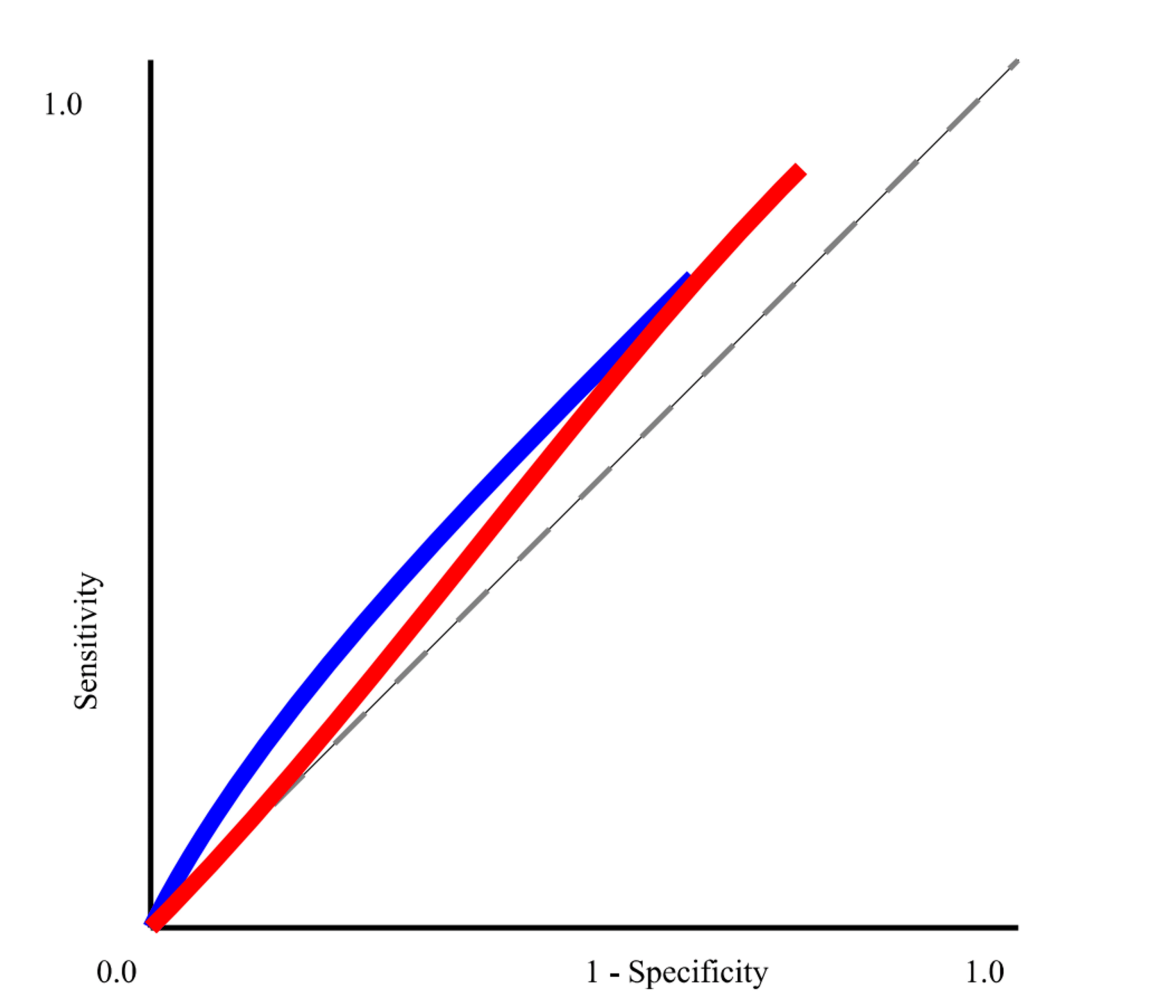
ROC analysis

The ROC curve (Figure 1) depicts the diagnostic efficacy of CRP and PCT in diagnosing sepsis, showcasing their capacity to differentiate between individuals with sepsis and those without. ROC curves graphically represent the relationship between sensitivity (the true positive rate) and 1-specificity (the false positive rate) at different threshold settings. The CRP curve (blue) has an AUC of 0.78, suggesting moderate diagnostic accuracy. It has a sensitivity of 85% and a specificity of 68%. By comparison, the PCT curve (red) exhibits a higher AUC value of 0.82, indicating superior diagnostic ability. It achieves a sensitivity of 88% and a specificity of 70%. These findings show that PCT is marginally superior than CRP in the diagnosis of sepsis, as evidenced by its greater AUC and improved balance between sensitivity and specificity.

**Figure 2:**
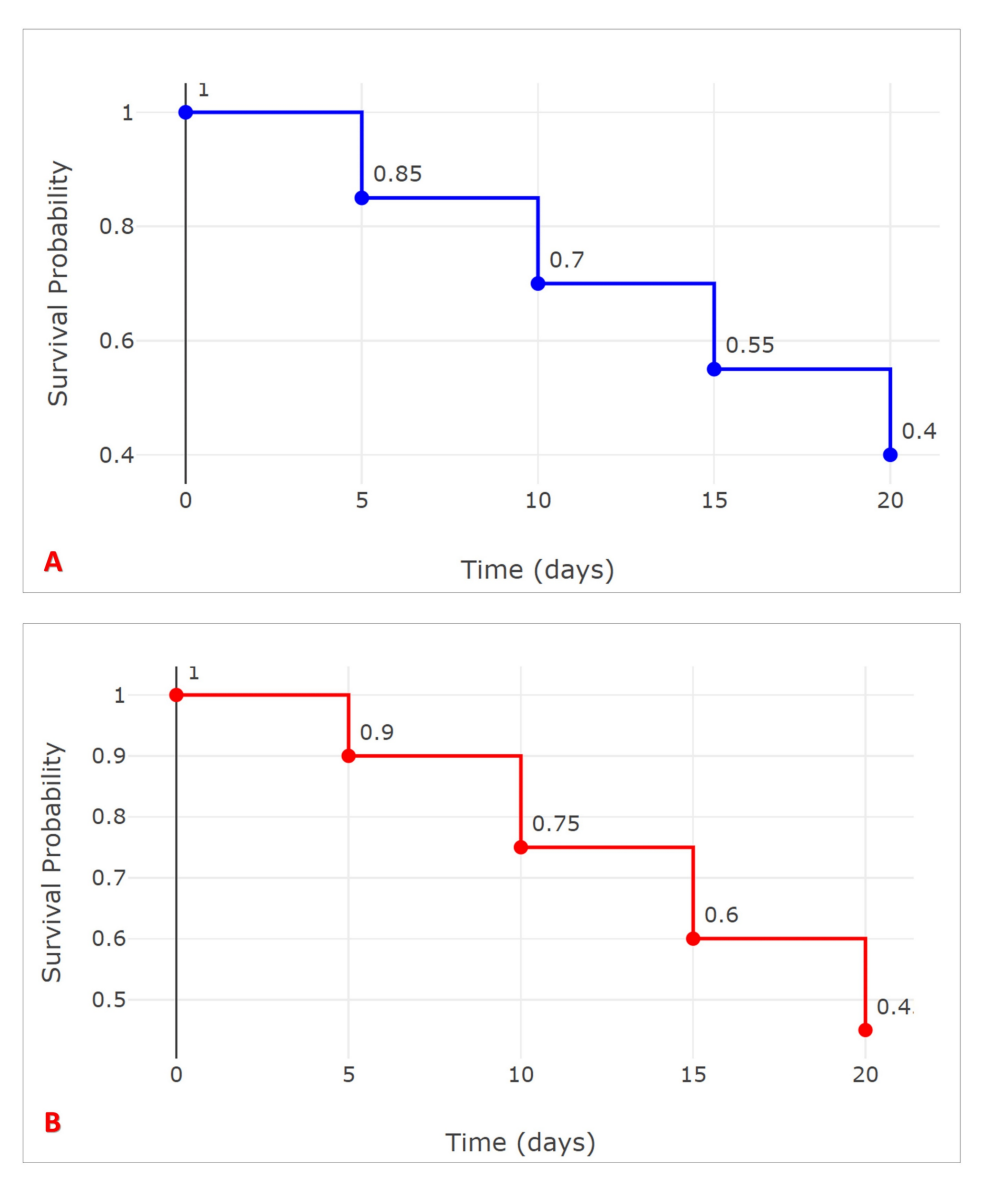
Kaplan-Meier curves for the CRP and PCT groups A: Kaplan-Meier curves for the CRP group; B: Kaplan-Meier curves for the PCT group CRP: C-reactive protein, PCT: procalcitonin

The Kaplan-Meier curves, as shown in Figure 2, illustrate the survival probabilities for the CRP and PCT groups over a 20-day period. In the CRP group, the survival probability decreases from 100% at day 0 to approximately 40% by day 20, indicating a significant decline in survival as time progresses. Conversely, the PCT group demonstrates a more gradual decline, with survival probabilities decreasing from 100% to around 45% over the same timeframe. These trends suggest that patients in the CRP group experience a higher rate of adverse events compared to those in the PCT group, emphasizing the potential prognostic value of CRP levels in predicting patient outcomes.

According to the Kaplan-Meier survival analysis (Table 2), participants with high CRP levels (>100 mg/L) had a median survival time of 18 days, which was significantly shorter than the 25 days for those with low CRP levels (≤100 mg/L), as indicated by a log-rank test p-value of 0.03. Similarly, those with high PCT levels (>2 ng/mL) had a median survival time of 15 days, significantly lower than the 27 days observed in the low PCT group (≤2 ng/mL), with a log-rank test p-value of 0.01. These results suggest that elevated CRP and PCT levels are associated with poorer survival outcomes in the study population, with p-values ≤0.05 indicating statistical significance.

**Table 2:** Kaplan-Meier survival analysis CRP (mg/L): C-reactive protein (mg/L); PCT (ng/mL): procalcitonin (nanograms per milliliter); p-value ≤ 0.05: significant

Group	Median survival time (days)	Log-rank test p-value	n = 90
High CRP (>100 mg/L)	18	0.03	45
Low CRP (≤100 mg/L)	25		
High PCT (>2 ng/mL)	15	0.01	45
Low PCT (≤2 ng/mL)	27		

The Cox proportional hazards regression analysis (Table 3) displays the association between several factors and the risk of death in the sample of 90 participants in the study. The analysis indicates that age, gender, and white blood cell count did not have a statistically significant impact on mortality, with p-values of 0.21, 0.47, and 0.15, respectively. A strong correlation was found between the Sequential Organ Failure Assessment (SOFA) score and mortality in both sepsis and septic shock groups. The hazard ratio (HR) for the SOFA score was 1.25 (95% CI: 1.10-1.42, p = 0.001) in the sepsis group and 1.30 (95% CI: 1.15-1.47, p = 0.001) in the septic shock group, indicating a 25% and 30% higher risk of death for each unit rise in the SOFA score, respectively. Elevated CRP levels (>100 mg/L) were associated with a 50% increased risk of death (HR: 1.50, 95% CI: 1.12-2.01, p = 0.008) in sepsis, while in septic shock, the risk increased to 70% (HR: 1.70, 95% CI: 1.20-2.32, p = 0.002). Similarly, PCT levels greater than 2 ng/mL were linked to a 68% higher risk in the sepsis group (HR: 1.68, 95% CI: 1.23-2.29, p = 0.002) and a 75% higher risk in the septic shock group (HR: 1.75, 95% CI: 1.30-2.35, p = 0.001). Lactate levels also correlated with mortality, with a hazard ratio of 1.22 (95% CI: 1.03-1.45, p = 0.02) in sepsis and 1.30 (95% CI: 1.05-1.60, p = 0.03) in septic shock. This model used females as the baseline group for the gender variable, enabling the assessment of the hazard ratio for males in relation to females. In this population, a p-value of <0.05 was deemed statistically significant, suggesting that the SOFA score, CRP, PCT, and lactate were significant predictors of death.

**Table 3:** Cox proportional hazards regression analysis (n = 90) *RG: "Female" is used as the reference group for the "Gender" variable, and the hazard ratio for "Male" shows how the risk of the outcome differs from that of the female baseline. p-value ≤ 0.05: significant; SOFA Score: Sequential Organ Failure Assessment Score; CRP (mg/L): C-reactive protein (mg/L); PCT (ng/mL): procalcitonin (nanograms per milliliter); WBC (×10⁹/L): white blood cell count (billions per liter); Lactate (mmol/L): Lactate (millimoles per liter)

Variable	Sepsis		Septic shock	
	Hazard ratio (HR)	p-value	Hazard ratio (HR)	p-value
Age (per year)	1.02	0.21	1.03	0.15
Male	1.15	0.47	1.1	0.42
Female	1.00 (RG)*	-	1.00 (RG)*	-
SOFA score	1.25	0.001	1.3	0.001
CRP (>100 mg/L)	1.5	0.008	1.7	0.002
PCT (>2 ng/mL)	1.68	0.002	1.75	0.001
WBC (×10⁹/L)	1.08	0.15	1.12	0.18
Lactate (mmol/L)	1.22	0.02	1.3	0.03

## Discussion

The findings of this study align with and build upon previous research on the prognostic significance of CRP, PCT, and other clinical markers in sepsis. The mean age of the study population (65.4 years) and the predominance of males (60%) are typically more affected by severe sepsis and septic shock. The observed SOFA score (mean: 7.2) reflects moderate organ dysfunction, which is a well-established predictor of poor outcomes in septic patients, corroborating findings from studies that have identified SOFA as a robust indicator of mortality risk [13].

The elevated mean CRP (102.3 mg/L) and PCT (5.4 ng/mL) levels in this cohort are in line with previous reports that have highlighted the role of these biomarkers in sepsis. Notably, the diagnostic performance of CRP and PCT, as evidenced by their respective AUCs of 0.78 and 0.82, confirms that PCT is slightly more reliable than CRP in diagnosing sepsis, a finding that has been consistently reported in recent studies [10,14]. This is particularly relevant given the ongoing debate regarding the most effective biomarkers for early sepsis detection.

The Kaplan-Meier survival analysis results further emphasize the prognostic value of CRP and PCT, with elevated levels of both markers associated with significantly shorter survival times. This association has been supported by studies [15,16], demonstrating that high CRP and PCT levels correlate with increased mortality in septic patients, underscoring their utility as prognostic indicators.

The Cox proportional hazards regression analysis revealed that elevated SOFA scores, CRP, PCT, and lactate levels were significant predictors of mortality. The hazard ratios of 1.25 for SOFA score, 1.5 for CRP, and 1.68 for PCT reinforce findings from previous studies that have identified these variables as independent predictors of mortality in sepsis [17-19]. Interestingly, while the white blood cell count did not emerge as a significant predictor in this study, it is often considered in conjunction with other clinical factors to assess the severity of infection, reflecting the complex and multifactorial nature of sepsis outcomes. These findings collectively validate the role of CRP, PCT, SOFA score, and lactate as critical tools in the early diagnosis and prognostication of sepsis, and they align well with contemporary research that underscores their clinical importance in managing this life-threatening condition.

Limitations

This study is subject to limitations, such as its relatively small sample size and single-center design, which may restrict the applicability of the results. Furthermore, due to the observational character of the study, it is not possible to demonstrate a causal relationship between biomarkers and outcomes. Nevertheless, a significant strength resides in the thorough examination of both CRP and PCT levels with clinical scores, such as SOFA, which amplifies the reliability of the prognostic observations. These findings emphasize the need of combining several biomarkers with clinical evaluations to effectively manage sepsis at an early stage. Future research should focus on doing studies with bigger groups of participants from many medical centers in order to confirm these findings and examine the possibility of integrating these biomarkers into established sepsis treatment guidelines.

## Conclusions

This study demonstrates that both CRP and PCT are valuable biomarkers for the early diagnosis and prognosis of sepsis in ICU patients. While PCT exhibits superior diagnostic performance compared to CRP, with a higher AUC of 0.82 versus 0.78, it is important to note that elevated PCT levels indicate the presence of bacterial infections rather than definitively confirming sepsis. High levels of both CRP and PCT are associated with increased mortality risk, highlighting their significance in early sepsis management. These results suggest the combined use of CRP and PCT in ICU settings to improve sepsis diagnosis, guide timely interventions, and ultimately enhance patient management and improve outcomes.
